# Parenting style patterns and their longitudinal impact on mental health in abused and nonabused adolescents

**DOI:** 10.3389/fpsyt.2025.1548549

**Published:** 2025-03-03

**Authors:** Wassilis Kassis, Aikaterini Vasiou, Dilan Aksoy, Céline Anne Favre, Sibylle Talmon-Gros Artz, Doug Magnusson

**Affiliations:** ^1^ Department of Research & Development, School of Education, University of Applied Sciences and Arts Northwestern Switzerland, Windisch, Switzerland; ^2^ Department of Primary Education, University of Crete, Rethymno, Greece; ^3^ School of Child and Youth Care, University of Victoria, Coast Salish Territories, Victoria, BC, Canada; ^4^ Department of Educational Psychology and Leadership Studies, University of Victoria, Coast Salish Territories, Victoria, BC, Canada

**Keywords:** parenting styles, adolescents, latent person-oriented methods, longitudinal research, family abuse, mental health

## Abstract

**Background:**

While the impact of parenting styles on adolescents’ mental health is well documented, no study has used latent person-oriented methods to analyze the effects of parenting style trajectories, experienced by physically abused and nonabused adolescents from early to middle adolescence, on mental health outcomes.

**Method:**

In this longitudinal study, we used latent transition analysis (LTA) to detect parenting patterns and their trajectories among 1,709 adolescents from 44 high schools in Switzerland across three data waves (2021-2023) by applying a multigroup comparison between physically nonabused and abused adolescents. Using multinomial regression, we tested the effects of the detected parenting patterns on adolescents’ mental health.

**Results:**

Along with the two known patterns, termed “supportive” and “negative” parenting, two new parenting patterns which we termed “absent” (low levels on all tested parenting styles) and “ambiguous” (middle to high levels on all tested parenting styles) emerged as playing a key role in the perceptions of adolescents with and without parental abuse experience longitudinally. These four patterns developed in diverse ways: Supportive parenting decreased for abused adolescents over time but remained stable for the nonabused adolescents. The absent parenting level was stable over time among abused adolescents when compared to the outcomes experienced by adolescents subjected to the negative parenting pattern. Furthermore, we found a remarkable decline in the number of nonabused adolescents in the absence pattern from Wave 1 to Wave 3. Further, we also found that abused adolescents reported more negative parenting than nonabused adolescents. Additionally, we found that supportive parenting was beneficial for adolescents’ mental health whereas negative, ambiguous, and absent parenting all had detrimental effects.

**Conclusions:**

These findings highlight the beneficial association of supportive parenting and the detrimental effects of negative, ambiguous, and absent parenting. This also suggests that we must consider a more complex approach that involves examining a blend of different parenting styles when analyzing adolescent mental health.

## 1 Introduction

When children develop mental health issues, addressing only intervention and treatment on an individual level is surely needed but not enough. We also have to establish efforts required to target the universal promotion of mental health among youth on the family level, with parenting styles being crucial for psychiatric epidemiology (1–3). A positive parenting style can effectively prevent social, emotional, and behavioral difficulties, including risk behaviors (4–6). The experience of a mental health issue causes distress not only for the child or adolescent and their families but also interrupts the developmental course of the child which may negatively affect adult functioning, personality, and well-being. For example, research has shown that 50% of all adults with a mental health issue had the onset of symptoms before age 14 (7), and almost 75% are already symptomatic by the age of 18 (8).

Parenting styles characterize parents’ typical strategies and responses (9) and influence lifelong attachment (10). Parenting styles also have an impact on adolescents’ mental health and have been the subject of extensive scientific research (e.g., 11–13). These styles have important implications for prevention and intervention programs that consider family contextual factors (14). Such programs provide actionable policy recommendations, such as accessible funding, effective training, and supportive conditions to expand evidence-based parenting programs and promote competent parenting on a large scale (15). For example, Panter‐Brick et al. (16) in their practitioner review highlight the importance of designing interventions that appeal to all stakeholders and decision-makers, including fathers, mothers, other key caregivers, program directors, and funding organizations. In addition, a recent evidence-based study by Sanders (17) underscores the need for parenting support programs to be gender-sensitive, culturally responsive, and aligned with the local context, taking into account relevant policies, available resources, cultural influences, funding opportunities, workforce availability, and their capacity for program implementation.

Strongly connected to attachment theory, parenting styles emphasize how our early experiences with attachment figures shape our understanding of relationships and ourselves. As children interact with their parents or primary caregivers, they develop working models of how relationships function. These models include expectations about how we should be treated by others, how we should behave in relationships, and what we deserve in terms of care and attention (10). Consequently, parenting styles have a lasting impact on individuals’ life trajectories. According to Baumrind (18, 19) and Maccoby and Martin (20) four primary parenting styles are identified in the literature: authoritarian, permissive, neglectful, and authoritative. Authoritarian describes strong control and demands with little warmth and responsiveness, characterized by strict rules and expectations and little flexibility or emotional support. In contrast, permissive parenting involves high levels of warmth and responsiveness with low levels of control and demands and is characterized by leniency, indulgence, and few boundaries or expectations. Neglectful parenting shows low levels of warmth and control and is characterized by a lack of engagement, emotional support, and guidance. Authoritative, on the other hand, is characterized by high levels of warmth and control, combines clear expectations and firm boundaries with emotional support and open communication, is associated with fewer mental health problems, and acts as a buffer. In contrast, authoritarian parenting s is associated with increased negative mental health outcomes in adolescence and young adulthood, particularly if harsh parenting methods, such as physical abuse, are involved (21, 22). Physical abuse has been linked to a range of negative mental health outcomes in several studies (e.g. 23). In Switzerland, there is still no law on non-violent parenting and education and thus no prohibition of physical abuse (24). Given that child maltreatment, such as parental physical abuse, has been reported as a major key risk factor for mental health problems (e.g., 25, 26), such as low self-esteem and self-determination levels, combined with high levels of dissociation and depression/anxiety, it is crucial not only to examine the predominant parenting styles and their effects on adolescents’ mental health but also to uncover the presence of various underlying patterns of parenting styles.

## 2 Parenting styles, adolescents’ mental health, and the Alabama parenting questionnaire

A great body of research has examined the relationship between parenting styles and adolescents’ mental health, with results being inconsistent to a certain extent. In a meta-analysis, Pinquart (27) highlighted the association between parenting styles and internalizing behavior problems in children and adolescents. Based on the analysis of 1015 studies, Pinquart (27) showed that harsh control, psychological control, authoritarian, and neglectful parenting styles, were associated with increased levels of internalizing symptoms such as anxiety and depression. Two other meta-analyses support this finding. In their first meta-analysis, McLeon and colleagues (28) highlight the link between parenting subdimensions and child depression, showing that parental warmth was associated with lower levels of depression and negative parenting subdimensions 29 higher levels of depression. Regarding child anxiety and parenting, the authors of the second meta-analysis showed that parental control was more strongly associated with child anxiety than parental rejection, although overall there was only a small association between parenting style and child anxiety (28). Further, adolescents’ self-determination is enhanced by positive parenting dimensions, especially the granting of autonomy (30). Positive parenting thus appears to be an exceptionally essential buffer and resilience factor against mental health problems. Pinquart (27) also examined the effect of parenting styles on the change in internalizing symptoms over time. High levels of parental warmth, behavioral control, autonomy granting, and authoritative parenting decreased the level of internalizing problem behaviors over time, while harsh control, psychological control, authoritarian, and neglectful parenting styles increased internalizing problems. Studies have shown that negative parenting, especially authoritarian parenting when connected to parental physical abuse, has a specific negative effect on the self-esteem of children and adolescents (31, 32), while positive parenting, characterized parenting warmth, shows positive impacts on self-esteem (29).

Tabak and Zawadzka (33), using the Alabama Parenting Questionnaire (APQ), examined positive parenting’s importance in predicting adolescents’ mental health as well as whether its positive effect will be discernible over the five years (ages 13 to 18 years). They observed a significant decrease in positive parenting practices and showed that positive parenting directly predicted children’s mental health. Given the above findings, it can be stated that parenting styles have a proven strong impact on adolescents’ mental health, and we have highlighted that parenting style is a multidimensional construct with complex associations with mental health outcomes. Studies have demonstrated that authoritarian parenting is a central indicator for understanding the socialization pathways of physically abused adolescents by their parents (34, 35). Interestingly, although the Alabama Parenting Questionnaire (APQ) is an important and frequently used scale for measuring parenting behavior, the absence of a sub-scale on authoritarian parenting within the APQ is deemed a shortcoming (32, 34). Therefore, in the current study, we used the evaluated and validated (e.g., 36–38) 9-item short form (39) of the APQ (38, 40), which encompasses scales for positive parenting, poor monitoring, and inconsistent discipline, and additionally included a scale for the authoritarian parenting style.

## 3 Parenting style profiles of physically abusive parents

Taking into consideration the internationally validated proliferation of 25-30% (26, 41) of parental physical abuse, we needed a more focused look at the respective parents’ parenting styles. Although parenting style profiles identified by person-centred approaches have been rarely studied for the presence of physical abuse (see 42), primarily variable-oriented research has shown that abusive parents tend to be less positive and less involved with their children (13, 43). Okado and Haskett (44) used a latent longitudinal person-oriented analytic approach to identify two latent trajectories: a larger class of parents with positive effects and a smaller, harsher class with negative effects. Because the parenting style patterns of abusive parents appear to differ from those of parents who do not abuse their children, it seems relevant to analyze these two groups separately. Although the severity of physical parental abuse is often debated (26), it is important to weigh “minor” forms (i.e., slapping a child’s face or hand) differently from more serious forms such as kicking and punching. The meta-analysis of Stoltenborgh et al. (45) concluded that when focusing on extreme forms of physical abuse, the prevalence, not the incidence of parental abuse provides the most helpful frame through which to view adolescents’ developmental processes and outcomes. Emphasizing the prevalence of these more extreme forms of physical parental abuse shows that even a single episode of physical abuse can contribute a great deal to adolescents’ mental health problems (13, 46).

## 4 Insights on parenting style patterns by applying person-centered approaches

Recent empirical studies (e.g., 47–49) increasingly recognize the multidimensionality and complexity of parenting styles. They emphasize the importance of considering patterns, that is a blend of different parenting styles when studying parenting behavior. These studies use a person-centered approach that enables researchers to examine various parenting types and the other risk and resilience factors that relate to and flow from parenting typologies. For example, Carpenter and Mendez (50) explored longitudinal parenting pattern differences in preschoolers’ behavioral adjustment by evaluating preschoolers’ aggression and hyperactivity in the fall and spring of the academic year. Bowers et al. (51) examined the role of youth-reported parenting style latent profiles in promoting positive youth development. Zhang et al. (52) examined Chinese maternal parenting styles as part of the subtypes and how they were stable or changing in the early adolescent period. Using the Self-Determination Theory (53), multigroup latent profile analyses showed that a high monitoring/high autonomy support parenting profile was associated with the best outcomes in adolescent adjustment, and a low monitoring/high psychological control parenting profile was associated with the worst adjustment outcomes.

Other longitudinal empirical studies using latent person-centred methods have shown that parenting styles do not always occur in pure form but that mixed forms among parents may reveal other patterns of parenting (54). For example, Kim et al. (55), in a three-wave longitudinal study lasting eight years, from early adolescence to emerging adulthood, identified four parenting profiles: supportive, tiger (harsh and focused on school success), easy-going, and harsh, in Chinese American families. In addition, Teuber et al. (56), using longitudinal latent person-oriented perspectives, found four autonomy-related parenting profiles (supportive, controlling, unsupportive-uncontrolling, and limited supportive). Furthermore, Bouffard and Labranche (57), analyzed a sample of 672 students to identify distinct profiles of parenting support of autonomy and control and investigated if membership in these profile groups relates to students’ school adjustment once they are in middle school. These researchers found a three-class/group model of parenting consisting of an autonomy-centered group, a control-centered group, and an autonomy/control-balanced group.

Thus, in comparison to variable-oriented approaches that consider parenting as one-dimensional and subject to situational or contextual variation, in this study, we aim to use a bottom-up approach to identify profiles that represent an underlying multidimensional construct. More precisely, we attempted to provide a richer characterization of parenting styles and their association with externalizing problems, internalizing problems, and prosocial behaviors of children and adolescents using latent profile analysis and examining possible relations between the derived profiles and such externalizing-internalizing-prosocial variables. Based on these findings, the present study treats parenting styles as a multidimensional latent construct that includes different dimensions of parenting instead of focusing on a single prominent parenting style.

## 5 Current study

Following these lines of thought, we wanted to find out whether and how the perceived parenting style patterns of physically abusive parents differ from those of non-abusive parents and how these patterns influence the mental health of physically abused and nonabused adolescents over time. To fill this research gap and raise the question of whether parenting style - like so many psychological constructs - is not a trait but a state and additionally has unique patterns of parental experience that deviate from standard models, it is crucial to track person-centered parenting styles longitudinally. To do this we used the APQ to answer the research question “Do parenting style patterns differ between physically abusive and non-physically abusive parents and how do these different parenting styles affect the mental health of physically abused and nonabused adolescents over time?” We hypothesized (H1) that we would identify distinct parenting patterns using latent-class analysis (LCA) and latent-transition analysis (LTA). We expected to find one class characterized by supportive parenting, one that identified mainly negative parenting, and one or two classes with more mixed patterns. When looking for longitudinal changes (H2), we expected the greatest stability in the negative parenting pattern and the lowest stability in the supportive parenting pattern for nonabused and abused adolescents, but we hypothesized (H3) that abused youths would more often be in the negative parenting pattern and less frequently in the supportive pattern than the nonabused adolescents. Finally, we expected that parenting patterns would display statistically significant differences in abused and nonabused adolescents’ mental health conditions (H4).

## 6 Methods

### 6.1 Study and participants

Data from 1,709 adolescents from seventh to ninth-grade classes in 44 high schools in Switzerland were collected as part of a three-wave longitudinal study with wave 1 in the spring of 2021 (mean age =M_age_12.28, SD = 0.56), wave 2 in the spring of 2022 mean age = 13.71, SD = 0.54), and wave 3 in the spring of 2023 (mean age = 14.26, SD = 0.54). Since the participants were not of legal age, they provided written assent, and their parents informed written consent to participation; no external incentives were offered. The Ethics Committee of the School of Education, University of Applied Sciences and Arts Northwestern Switzerland approved the study (reference number: 040620).

### 6.2 Measures

#### 6.2.1 Measuring the prevalence of parental physical abuse for multigroup analysis

As already referred and following the meta-analysis of Stoltenborgh et al. (45) we focused on the prevalence of extreme forms of physical abuse and did not include the APQ-subscale on corporal punishment because it consists of very different physical abuse forms such as slapping, spanking, and hitting. We applied a single-item indicator measuring the prevalence of parental physical abuse, with participants reporting whether they had experienced severe forms of physical abuse at least once in their lifetime. Responses were dichotomized as no (0) or yes (1).

#### 6.2.2 Parenting styles

To measure parenting styles, we used the 42-item APQ (37) which includes subscales for parental involvement, positive parenting, poor supervision, inconsistent discipline, and physical punishment. We applied two adaptations: First, we excluded the APQ subscale for corporal punishment from the LCA and LTA for the modeling steps, because we used the multigroup model to differentiate between physically abused and nonabused adolescents. We chose this step due to our modeling process, since we used multi-group analysis to distinguish between participants with and without physical abuse experiences. To include the corporal punishment subscale might have interfered with the results as it directly relates to the criteria for subgroup definition. By omitting the subscale, we ensured that the differentiation of the subgroups was not influenced by overlapping constructs, thus influencing validity. In their recent study, Florean et al. (58) based on the confirmatory analyses of Esposito et al. (59) and Święcicka et al. (60) also mentioned that the scale of corporal punishment should be considered independently. In the same study, seven supplementary items were used to avoid a bias toward the items that measure corporal punishment with APQ (38). Second, following the proposal of Shaffer et al. (61), that research could use different types of parenting measures and could combine behavior-focused tools like the APQ and measures of parenting knowledge, values, and goals, we added one additional parenting style dimension, the authoritarian parenting style (18, 1968), a seven-item dimension, that was missing in the APQ.

All parenting dimensions were rated on a 5-point Likert scale (1 = never to 5 = always), Mean scores for the respective five dimensions were calculated, all responses were median split for LCA/LTA. We included the following five parenting dimensions (see **Table 1** for Cronbach’s *a* and Median-Split values): (a) parental involvement, using nine items (e.g., “You have a friendly talk with your mom/dad”, “My mother/My father helped me with my homework”; (b) positive parenting, using three items (e.g., “Your parents tell you that you are doing a good job”, “My parents hug or kiss me when I doing something well”; (c) poor monitoring/supervision, using five items (e.g., “You stay out in the evening past the time you are supposed to be home”, “I am out with friends my parents don’t know”; (d) inconsistent parenting, using three items (e.g., “ Your parents threaten to punish you and then do not do it”, “I am talking my parents out of being punished after I have done something wrong”; and (e) authoritarian style, using seven items (e.g., “My parents expect me to comply with everything they tell me to do”, “My parents often don’t tolerate any contradiction.”;. Items assessing the first two constructs are worded positively, and items assessing the latter three constructs are worded negatively.

**Table 1:** Parenting style cronbach’s *a* and median split values.

	Cronbach’s *a* (Median Split)		
	Wave 1	Wave 2	Wave 3
Parental Involvement	.85 (3.33)	.86 (3.22)	.87 (3.22)
Positive Parenting	.81 (4)	.81 (4)	.82 (3.66)
Poor Monitoring	.72 (2)	.73 (2)	.74 (2.22)
Inconsistent Parenting	.67 (2)	.69 (2)	.71 (2)
Authoritarian Style	.81 (2)	.84 (2)	.87 (2)

#### 6.2.3 Measures of mental health outcomes

Self-esteem was assessed using the Rosenberg Self-Esteem Scale (62), which consists of a 5-item short form (Cronbach’s α_ w1 = .92, Cronbach’s α_wave2 = .93, Cronbach’s α_wave3 = .92) rated on a 4-point Likert scale (1 = not true to 4 = completely true).

Anxiety and depression symptoms were examined using the modified version of the Hopkins Symptom Checklist (63). Participants rated 24 items on a 4-point Likert scale (1= not at all to 4 = extremely; Cronbach’s α_wave1 = .96, Cronbach’s α_wave2 = .96, Cronbach’s α_wave3 = .97. We deleted one item about sexuality from the 25-item version because of the respondents’ age.

Dissociation was evaluated using the four-item Dissociation Tension Scale Acute [(64); 1 = not at all to 4 = very much] measuring depersonalization, somatoform, derealization, and analgesia (Cronbach’s α_wave1 = .85, Cronbach’s α_wave2 = .86, Cronbach’s α_wave3 = .87).

Self-determination was measured based on Deci and Ryan’s (65) measures of basic psychological needs, autonomy, competence, and relatedness, with three items each. The nine-item scale (Cronbach’s α_wave1 = .90, Cronbach’s α_wave2 = .92, Cronbach’s α_wave3 = .93) used a 4-point Likert scale (1 = not true at all to 4 = completely true).

#### 6.2.4 Covariates

Gender was derived from the class rosters provided by the teachers. Students’ socio-economic background (SES) was calculated by aggregating four indicators: the education level of each student’s mother and father and an estimate of the number of their and the students’ books (66). Migration background (MB) was obtained by determining whether the students and their parents had only Swiss nationality and were born in Switzerland.

### 6.3 Analytic plan

First, we conducted the descriptive statistical analysis described above. Second, to examine the structure of parenting styles, we ran a confirmatory factor analysis for all three waves and the five parenting style dimensions for abused and nonabused participants. Third, we explored parenting style patterns cross-sectionally by conducting LCA for all three waves separately and then by LTA longitudinally by conducting a multigroup comparison between nonabused and abused adolescents. Fourth, we applied an invariance analysis across abuse experiences to ensure the reliability for the identified number of parenting (configural invariance) as well as the same relevance of the parenting patterns (metric invariance) for both abused and nonabused adolescents. Fifth, we tested the associations of parenting styles with adolescents’ mental health by multinomial regression. LCA/LTA models were estimated with Mplus (67). All other computations were completed using SPSS 25.

### 6.4 Results

#### 6.4.1 Descriptives

We ran t-tests (see **Table 2**) to analyze wave-specific mean differences in sociodemographic variables between the nonabused and abused subsamples. No gender differences were displayed (see **Table 2**). However, there were significantly higher percentages of adolescents with an MB and a lower SES in the abuse subsamples than in the nonabused sample for all three study waves. Still, that relevance should not be overestimated because the respective Cohens’d were very low.

**Table 2:** Wave-specific sample means (and standard deviations) for the longitudinal sample for the Nonabused (n=1,146) and Abused (n=563) subsamples.

	Wave 1			Wave 2			Wave 3		
	Nonabused	Abused	d	Nonabused	Abused	d	Nonabused	Abused	d
Gender % Female	1.50 (.50) 47.7	1.53 (.49) 48.9	–	1.51 (.50) 48	1.52 (.50) 48.4	–	1.49 (.51) 47.4	1.56 (.52) 50.4	–
MB	.52 (.50)	.67*** (.47)	-.31	.56 (.49)	.77*** (.42)	-.45	.46 (.50)	.61*** (.49)	-.30
% MB	51.7	67.1		55.7	77.1		46.1	60.7	
SES	1.98 (.63)	1.89** (.64)	.15	1.96 (.63)	1.85** (.64)	.16	1.99 (.63)	1.87*** (.65)	.19
Low (%)	21.1	26.6		22.1	28.4		20.0	28.5	
Middle (%)	59.7	58.0		59.5	57.3		60.6	55.9	
High (%)	19.2	15.4		18.4	14.3		19.4	15.6	

MB, Migration background; SES, Socio-economic background; **p <.01; ***p <.001.; d = Cohen’s d is reported when the results are significant.

Related to sample attrition from Wave 1 to Wave 3, we consider the three samples comparable because we only noticed a significant difference for MB, t(1,709) = 5.69, p <.001, with a very low Cohen’s d (d = .14) but no significant differences for gender, t(1,709) = .506, p = .660, or SES, t(1,709) = .623, p = .533.

Second (see **Table 3**), we identified higher levels of poor supervision from Wave 1 to Wave 2 for both subsamples but no significant changes from Wave 2 to 3. Higher levels of inconsistent and authoritarian parenting were only detected for abused participants from Wave 1 to Wave 2. Positive parenting levels decreased for all samples from Wave 1 to Wave 2 and increased from Wave 2 to Wave 3.

**Table 3:** Means and standard deviations of dichotomized LCA/LTA for the nonabused (n=1,146) and abused (n=563) subsamples.

Variables	Wave 1 Nonabused	Wave 2 Nonabused	Wave 2 Nonabused	Wave 3 Nonabused	Wave 1 Abused	Wave 2 Abused	Wave 2 Abused	Wave 3 Abused
	M (SD)	M (SD)	M (SD)	M (SD)	M (SD)	M (SD)	M (SD)	M (SD)
Involvement	1.62 (.49)	1.65 (.48)*	1.65 (.48)	1,61 (.49)*	1.38 (.49)	1.37 (.48)	1.37 (.48)	1.40 (.49)
Positive	1.47 (.50)	1.42 (.49)***	1.42 (.49)	1.61 (.49)***	1.32 (.47)	1.22 (.41)***	1.22 (.41)	1.40 (.49)***
Poor Supervision	1.36 (.48)	1.43 (.49)***	1.43 (.49)	1.43 (.49)	1.48 (.50)	1.59 (.49)***	1.59 (.49)	1.58 (.49)
Inconsistent	1.38 (.49)	1.41 (.49)	1.41 (.49)	1.43 (.49)	1.59 (.49)	1.66 (.47)**	1.66 (.47)	1.61 (.49)
Authoritarian	1.37 (.48)	1.35 (.48)	1.35 (.48)	1.37 (.48)*	1.63 (.48)	1.70 (.46)*	1.70 (.46)	1.67 (.47)

*** = p <.001, ** = p <.01, * = p <.05

Paired samples t-test to the respective next wave.

#### 6.4.2 Confirmatory factor analysis

There were acceptable fits (see **Table 4**) for all three waves for the overall sample and for both subsamples (nonabused and abused adolescents).

**Table 4:** Results of confirmatory factor analysis for the different samples (overall, multigroup, nonabused, and abused) for each of the three waves.

Sample	df	Chi2	p-Value	RMSEA	SRMR	CFI	TLI
Wave1_overall	291	1013.853	<.001.	.036	.043	.955	.946
Wave1_multigroup	626	1173.846	<.001.	.033	.046	.955	.950
Wave1_nonabused	291	766.345	<.001.	.033	.040	.959	.950
Wave1_abused	291	444.662	<.001.	.032	.049	.962	.955
Wave2_overall	291	1221.519	<.001.	.043	.047	.943	.931
Wave2_ multigroup	626	1649.921	<.001.	.044	.056	.932	.923
Wave2_nonabused	291	1079.576	<.001.	.042	.046	.944	.933
Wave2_abused	291	608.900	<.001.	.044	.054	.933	.919
Wave3_overall	291	1281.725	<.001.	.046	.055	.940	.927
Wave3_ multigroup	626	1600.469	<.001.	.047	.062	.932	.924
Wave3_nonabused	291	1102.401	<.001.	.046	.055	.939	.926
Wave3_abused	291	639.720	<.001.	.052	.070	.928	.913

**Figure 1:**
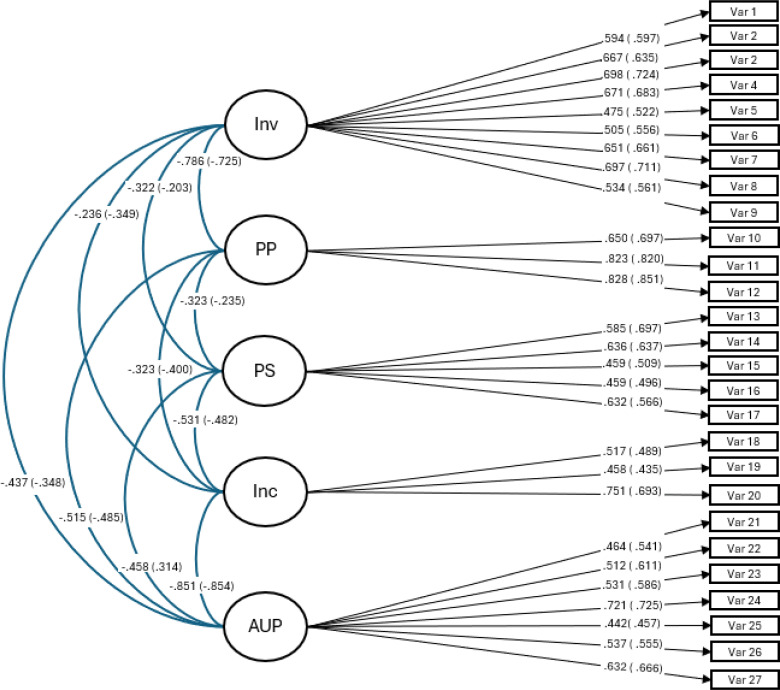
Factor loadings, wave 1, for the five-factor parenting styles, 27-item model. inv, involvement; pp, positive parenting; ps, poor supervision; inp, inconsistent parenting; aup, authoritarian parenting. Without brackets are the specific values for the nonabused sample and with brackets are the values of the abused sub-sample.

**Figure 2:**
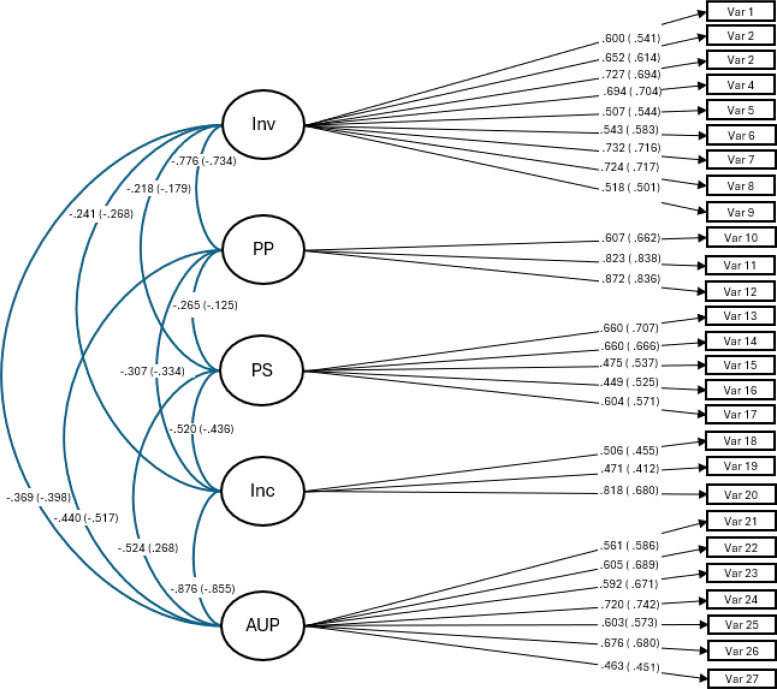
Factor loadings, wave 2, for the five-factor parenting styles, 27-item model. inv, involvement; pp, positive parenting; ps, poor supervision; inp, inconsistent parenting; aup, authoritarian parenting. Without brackets are the specific values for the nonabused sample and with brackets are the values of the abused sub-sample.

**Figure 3:**
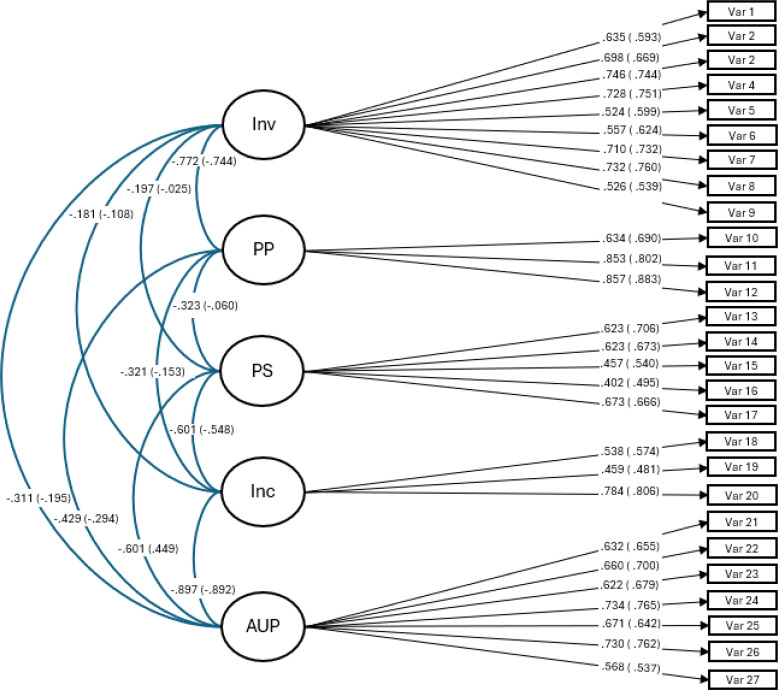
Factor loadings, wave 3, for the five-factor parenting styles, 27-item model. inv, involvement; pp, positive parenting; ps, poor supervision; inp, inconsistent parenting; aup, authoritarian parenting. Without brackets are the specific values for the nonabused sample and with brackets are the values of the abused sub-sample.

The RMSEA, TLI, and CFI are deemed particularly important for accurately estimating CFAs (68). Following Marsh et al. (69), we established a satisfactory model fit as RMSEA values below.08, coupled with CFI and TLI values above.90 and SRMR values below.08, indicating a strong fit for the model. The fit indices obtained from the confirmatory factor analysis applied were strong for all three parenting style scales and the different samples (see **Table 4**). The initial standardized factor loadings (see **Figures 1**–**3**) for the five-factor, 27-item model, for all three waves and both, the nonabused as the abused adolescents are an additional confirmation for the construct validity of the study’s scales.

#### 6.4.3 Exploring parenting patterns cross-sectionally with multigroup LCAs and longitudinally with LTAs

LCA/LTA are person-centred approaches focusing on latent classes and trajectories, which are determined by categorical indicators (70). LCAs were conducted for cross-sectional and LTAs for longitudinal data. LCA/LTA models were estimated for two to six latent classes on five parenting styles to define the most parsimonious model. The determination of an LCA or LTA model was conducted based on statistical criteria and theoretical frameworks (71). The focus of our study was to determine significantly distinct parenting classes using statistical indices to determine the optimal number of latent classes: AIC, aBIC, and significant LMR, aLMR, and BLRT (72). We conducted LCAs and LTAs while applying multigroup analyses exploring parenting style patterns for both subsamples. Following Lanza et al. (70) and Lanza & Cooper (72) the chosen model for the applied LCAs and LTAs (i.e., how many classes) was based on a mix of statistical indicators and extant theoretical considerations.

First, the parenting style classes were studied through separate multigroup LCAs for all three waves using five measures on parenting practice domains (parental involvement, positive parenting, poor monitoring/supervision, inconsistent parenting, and authoritarian style). The four-class solution was identified (see **Table 5** on LCA model fits) as having lower aBIC-values compared to the three-class and the five-class solutions. Another argument for the four-class solution compared to the three-class solution was that it gave additional insights into differential processes in parenting patterns by the fourth identified class. Compared to the five-class solution, the four-class solution was the most parsimonious model, as the five-class solution did not offer new theoretical insights (see **Table 5**). All separate comparisons for all three waves between the chosen four-class and the three-class or five-class solutions validated the selection in favor of the four-class solution. The very high classification accuracy was an additional confirmation that the chosen four-class solution fits both subsamples.

**Table 5:** Latent class multigroup analysis model fit statistics to select the number of classes for all three waves sequentially.

	AIC	BIC	aBIC	Entropy	Sample proportion in % per class	Classification accuracy
Wave 1 2-Profiles	11829	119f46	11877	.609	38.3;18.2/29;14.0	.875-.895
3-Profiles	11713	11890	11785	.722	39.3;18,8/10.8;5.0/17,6;8.4	.719-.934
4-Profiles	11684	11920	11780	.709	9.1;4.3/32.6;15.5/9.4;4.4/16.6;7.9	.729-.888
5-Profiles	11694	11989	11815	.657	13.3;6.4/8.4;4.0/15.2;7.3/22.9;10.9/7.7;3.6	.650-.819
6-Profiles	11710	12064	11855	.689	10.6;5.1/19.2;9.1/3.1;1.5/9.4;4.4/7.6;3.6/17.6;8.4	.698-.815
Wave 2 2-Profiles	12721	12841	12771	.523	37.2;18.5/29.7;14.6	.853-.858
3-Profiles	12520	12700	12595	.677	19.4;9.6/18.3;8.9/29.2;14.5	.840-.865
4-Profiles	12489	12729	12589	.670	13.5;6.6/18.9;9.3/25.8;12.8/8.7;4.3	.805-.853
5-Profiles	12503	12802	12627	.648	14.8;7.3/8.0;3.9/25.5;12.6/2.1;1.1/16.4;8.1	.530-.810
6-Profiles	12520	12879	12669	.664	11.6;5.7/8.0;3.9/2.6;1.2/12.8;6.3/25.4;12.6/6.4;3.1	.638-.792
Wave 3 2-Profiles	10506	10622	10552	.623	33.1;15.3/35.1;16.5	.888-.899
3-Profiles	10329	10502	10397	.715	18.9;9.2/31.3;13.9/17.9;8.6	.863-.897
4-Profiles	10289	10521	10381	.713	26.2;12.2/14.1;6.5/19.4;8.9/8.3;4.1	.787-.912
5-Profiles	10301	10590	10415	.747	27.4;12.8/3.3;1.6/20.9;9.7/9.7;4.3/6.9;3.3	.702-.886
6-Profiles	10315	10662	10452	.747	27.4;12.6/13.3;6.2/4.5;2.1/9.1;4.3/8.2;3.8/5.6;2.6

AIC, Akaike information criterion; BIC, Bayesian information criterion; aBIC, adjusted Bayesian information criterion.

Secondly, by conducting multigroup LTAs, we explored whether the same latent status could be identified in all three waves and detected the four-class solution as the best (for model fits, see **Table 6**). From three to four classes, we split a bigger class into two middle size classes and gained insights into differential parenting processes. The aBIC also dropped (see **Table 6**) in the multigroup LTA between the three- and four-class solutions. In comparison to the three-class solution, the aBIC drop to the four-class solution was substantial (Δ-554), and the respective drop from the four-class to the five-class only minimal (Δ-122), indicating a clear elbow effect (see **Table 6**), and the four-class solution was therefore the appropriate one whereas the five-class solution did not offer any substantial content knowledge.

**Table 6:** Latent transition multigroup analysis model fit statistics to select longitudinally the number of classes over three waves.

Classes	AIC	BIC	aBIC	Entropy Wave 1/Wave2/Wave3	Samples in %	Classification Accuracy
2	30796	30905	30841	.671/.737/.684	W1:46.8/53.2 W2: 51.1/48.9 W3: 49.4/50.6	W1:.897-.910 W2:.920-.926 W3:.903-.915
3	29880	30098	29971	.699/.744/.690	W1: 25.7/40.8/33.4 W2: 26.0/36.1/37.9 W3: 20.9/40.8/38.3	W1:.843-. 886 W2:.852-. 909 W3:.805-. 890
**4**	**29267**	**29267**	**29417**	**.681/.742/.686**	**W1: 24.2/31.1/25.2/19.3 W2: 24.9/24.6/26.5/23.9 W3: 23.4/29.1/21.2/26.2**	**W1:.774-.869 W2:.827-.895 W3:.779-.875**
5	29073	29606	29295	.695/.742/.688	W1: 20.1/16.9/11.7/28.2/22.9 W2: 17.6/20.6/15.8/22.1/23.7 W3: 12.0/23.6/13.5/28.2/22.6	W1:.765-.867 W2:.797-.886 W3:.719-.853
6	28924	29665	29233	.754/.796/.733	W1: 23.4/15.9/11.6/9.1/13.9/25.8 W2: 23.7/19.5/10.3/12.4/15.7/18.3 W3: 20.9/23.2/12.7/10.4/4-8/27.7	W1:.737-. 921 W2:.777-. 927 W3:.732-. 859

AIC, Akaike information criterion; BIC,Bayesian information criterion; aBIC, adjusted Bayesian information criterion; the chosen solution is highlighted in bold.

For all three waves (see **Tables 7**–**9**), we identified a “supportive parenting” class with high levels of involvement and positive parenting, a class called “negative parenting” with high levels of poor supervision with inconsistent and authoritarian parenting, a class we called “ambiguous” because all parenting levels were elevated, and an “absent parenting” class coming close to parental neglect of the needed socio-emotional guidance of their children (43). Teuber et al. (56) called a similar class “unsupportive-uncontrolling” because all levels were low.

**Table 7:** Wave 1 patterns standardized coefficients: results in probability scale, % category high of the respective parenting style for the nonabused (n = 1,146), and abused (n = 563) subsamples of adolescents.

Wave	1	1	1	1	1	1	1	1
Sub-sample	nonabused	nonabused	nonabused	nonabused	abused	abused	abused	abused
pattern	supportive	negative	ambiguous	absent	supportive	negative	ambiguous	absent
Involvement	.857	.000	1.000	.267	.811	.191	.912	.257
Positive	.784	.153	.530	.004	.788	.047	1.000	.209
Poor Supervision	.215	.592	.509	.435	.021	.618	.563	.445
Inconsistent	.150	.929	1.000	.195	.102	.872	.654	.272
Authoritarian	.118	.893	.623	.424	.232	.883	.755	.302

**Table 8:** Wave 2 patterns standardized coefficients: results in probability scale, % category high of the respective parenting style for the nonabused (n = 1,146), and abused (n = 563) subsamples of adolescents.

Wave	2	2	2	2	2	2	2	2
Sub-sample	nonabused	nonabused	nonabused	nonabused	abused	abused	abused	abused
pattern	supportive	negative	ambiguous	absent	supportive	negative	ambiguous	absent
Involvement	.950	.152	.851	.298	.967	.000	.607	.000
Positive	.725	.012	.516	.112	.411	.000	.419	.215
Poor Supervision	.222	.721	.584	.491	.352	.689	.682	.531
Inconsistent	.171	1.000	.960	.076	.245	.834	1.000	.102
Authoritarian	.094	.818	.653	.279	.397	.892	.833	.326

**Table 9:** Wave 3 patterns standardized coefficients: results in probability scale, % category high of the respective parenting style for the nonabused (n = 1,146), and abused (n = 563) subsamples of adolescents.

Wave	3	3	3	3	3	3	3	3
Sub-sample	nonabused	nonabused	nonabused	nonabused	abused	abused	abused	abused
pattern	supportive	negative	ambiguous	absent	supportive	negative	ambiguous	absent
Involvement	.867	.212	.786	.314	.716	.000	.752	.284
Positive	.986	.079	.823	.196	1.000	.000	.688	.000
Poor Supervision	.234	.729	.535	.480	.364	.607	.798	.377
Inconsistent	.111	1.000	.960	.227	.152	.781	1.000	.000
Authoritarian	.081	1.000	.569	.351	.302	.861	.829	.285

We also identified (see **Table 10**) a heterogeneous development of the respective patterns with three distinct changes and an increase over time for the ambiguous pattern. Additionally, we noted a large decrease in the supportive pattern for abused adolescents. Further, we noticed a remarkable stability in the absent pattern for the abused adolescents along with a noteworthy decrease for the nonabused adolescents. Also, the supportive pattern was identified for fewer abused than nonabused adolescents in Wave 3, while for the nonabused, the negative pattern was smaller. Finally, for both subsamples, the “ambiguous” and “absent” patterns in Wave 3 were associated with one in four adolescents.

**Table 10:** Distributions of the four parenting patterns with the longitudinal overall sample (N = 1,709), nonabused (n = 1,146), and abused (n = 563) subsamples of adolescents over three waves by latent-transition analysis.

Parenting Style	Wave 1			Wave 2			Wave 3			ΔW3-W1 Changes in the respective sample		
	Overall	Nonabused	Abused	Overall	Nonabused	Abused	Overall	Nonabused	Abused	Overall in %	Nonabused in %	Abused in %
Supportive	31.0 (531)	41.0 (470)	10.8 (61)	24.5 (418)	34.3 (393)	4.4 (25)	28.8 (493)	39.0 (447)	8.2 (46)	−9.7	-4.9	-24.1
Negative	25.0 (427)	14.1 (162)	47.1 (265)	24.8 (423)	12.4 (142)	49.9 (281)	23.5 (402)	13.2 (151)	44.6 (251)	−6.0	-6.4	-5.3
Absent	24.7 (422)	26.7 (306)	20.6 (116)	26.8 (458)	29.3 (336)	21.7 (122)	21.7 (371)	22.3 (256)	20.4 (115)	-12.2	-16.5	-1.0
Ambiguous	19.3 (329)	18,2 (208)	21.5 (121)	24.0 (410)	24.0 (275)	24.0 (135)	25.9 (443)	25.5 (292)	26.8 (151)	+34.2	+40.1	+24.6

Note 1: Top number in each cell is the percentage. Bottom number in each cell is the frequency.

Note 2: Percentages add to 100 in columns.

#### 6.4.4 Invariance testing

By determining the optimal number of classes separately at each time point to be four (see LCA analysis), we validated for all three waves by separate LCAs the same number of classes (configural invariance). After that, we performed an LTA to estimate the probabilities of parenting pattern transitions over time from one latent class to another (Lanza et al., 2013). The LTA statistical step additionally explored whether the same latent patterns could be identified in all three waves and tested if the conditional response probabilities had been constrained to be metric invariant across abuse experiences and gender.

We tested for physical abuse measurement invariance by applying a Satorra-Bentler (73) corrected chi2 test corrected chi2 test for MLR, first on configural invariance across abuse experiences (Δchi2 [9] = 3.82, p > 0.05.) establishing the same number of parenting patterns in both subsamples. Secondly, we also tested whether the factor loadings (metric invariance) on the respective four patterns were the same and detected for abuse experiences the expected significant chi-square difference tests (Δchi2 [20] = 42.77, p < 0.001.) as well as for gender (Δchi2 [20] = 53.85, p < 0.001.). That means, that even if the number of parenting patterns is the same, the loadings, that is, the weight of the five indicators within the patterns depend on abuse experiences and gender.

#### 6.4.5 Association of the five parenting patterns with adolescents’ mental health

We ran multinomial regressions with three samples, the overall sample (N = 1,709), the subsample of the nonabused adolescents (n = 1,146), and the abused adolescents’ subsample (n = 563). The dependent variables were self-esteem, self-determination, dissociation, and anxiety/depression (see **Table 11**).

**Table 11:** Multinomial Logistic Regression of Mental Health Outcomes in the Four Parenting Patterns, for LTA Wave 3, with the longitudinal overall sample N=1,709, the Nonabused (n=1,146) and Abused (n=563) subsamples.

Mental Health Outcomes		Self-esteem				Self-determination				Dissociation				Anxiety/Depression			
Latent Class	sample	% Cox & Snell	OR [95% CL]	Wald	p	% Cox & Snell	OR [95% CL]	Wald	p	% Cox & Snell	OR [95% CL]	Wald	p	% Cox & Snell	OR [95% CL]	Wald	p
Reference is Supportive Parenting1																	
Negative	overall	6.8	.21 [.16,.29]	97.75	<.001	6.7	.24 [.18,.32]	90.09	<.001	8.8	5.87 [4.34, 7.93]	132.66	<.001	11.4	7.61 [5.61, 10.32]	170.51	<.001
	nonabused	4.2	.34 [.23,.51]	27.26	<.001	5.2	.29 [.19,.43]	37.11	<.001	6.4	5.04 [3.35, 7.59]	60.21	<.001	8.3	5.78 [3.86, 8.66]	72.27	<.001
	abused	8.3	.16 [.08,.32]	26.96	<.001	4.9	.27 [.14,.52]	14.70	<.001	5.8	4.92 [2.44, 9.93]	19.84	<.001	6.5	5.61 [2.86, 11.02]	25.11	<.001
Ambiguous	overall	6.8	.50 [.37,.70]	17.08	<.001	6.7	.67 [.49,.91]	6.40	.011	8.8	2.42 [1.78, 3.28]	32.24	<.001	11.4	2.35 [1.73, 3.19]	29.81	<.001
	nonabused	4.2	.85 [.55, 1.31]	.54	.461	5.2	.77 [.50, 1.18]	1.46	.227	6.4	2.33 [1.51, 3.58]	14.83	<.001	8.3	2.15 [1.41, 3.29]	12.65	<.001
	abused	8.3	.32 [.19,.53]	18.89	<.001	4.9	.68 [.42, 1.12]	2.25	.133	5.8	1.96 [1.22, 3.16]	7.67	<.001	6.5	1.68 [1.02, 2.76]	4.19	.041
Absent	overall	6.8	.38 [.28,.52]	36.95	<.001	6.7	.41 [.31,.56]	34.11	<.001	8.8	2.58 [1.93, 3.46]	40.45	<.001	11.4	2.62 [1.96, 3.52]	41.40	<.001
	nonabused	4.2	.57 [.38,.88]	6.59	.574	5.2	.48 [.32,.74]	11.31	<.001	6.4	2.33 [1.53, 3.56]	15.54	<.001	8.3	2.08 [1.37, 3.14]	11.99	<.001
	abused	8.3	.30 [.19,.49]	24.01	<.001	4.9	.39 [.25,.61]	17.16	<.001	5.8	2.50 [1.62, 3.85]	17.11	<.001	6.5	2.74 [1.76, 4.27]	19.84	<.001
Reference is Ambiguous Parenting2																	
Supportive	overall	6.8	1.98 [1.43, 2.74]	17.08	<.001	6.7	1.5 [1.09, 2.05]	6.41	.011	8.8	.41 [.30,.56]	32.24	<.001	11.4	.43 [.31,.58]	29.81	<.001
	nonabused	4.2	1.18 [.76, 1.82]	.54	.461	5.2	1.31 [.85, 2.02]	1.46	.227	6.4	.43 [.28,.66]	14.83	<.001	8.3	.46 [.30,.71]	12.65	<.001
	abused	8.3	3.16 [1.88, 5.30]	18.90	<.001	4.9	1.46 [.89, 2.40]	2.25	.113	5.8	.51 [.32,.82]	7.67	.006	6.5	.59 [.36,.98]	4.19	.041
Negative	overall	6.8	.42 [.32,.57]	34.05	<.001	6.7	.36 [.27,.48]	47.52	<.001	8.8	2.43 [1.81, 3.27]	34.49	<.001	11.4	3.24 [2.42, 4.35]	61.56	<.001
	nonabused	4.2	.40 [.29,.56]	29.08	<.001	5.2	.37 [.27,.52]	33.66	<.001	6.4	2.17 [1.55, 3.04]	20.18	<.001	8.3	2.68 [1.92, 3.75]	33.20	<.001
	abused	8.3	.50 [.24, 1.03]	3.57	.059	4.9	.39 [.19,.81]	6.40	.011	5.8	2.51 [1.19, 5.30]	5.83	.016	6.5	3.33 [1.62, 6.86]	10.77	<.001
Absent	overall	6.8	.76 [.56, 1.01]	3.47	.062	6.7	.62 [.46,.83]	10.31	<.001	8.8	1.07 [.80, 1.42]	.21	.648	11.4	1.12 [.84, 1.48]	.60	.439
	nonabused	4.2	.68 [.47,.97]	4.63	.031	6.7	.63 [.44,.90]	6.33	.012	6.4	1.00 [.70, 1.43]	.00	.989	8.3	.96 [.68, 1.36]	.05	.831
	abused	8.3	.95 [.57,1.59]	.04	.852	4.9	.58 [.34,.97]	4.36	.037	5.8	1.27 [.77, 2.11]	.88	.348	6.5	1.63 [.98, 2.72]	3.54	.060

OR, Odds Ratio. 1Reference LTA wave 4 pattern is “Supportive Parenting” with high levels of involvement and positive parenting, low levels of poor supervision, inconsistent, and authoritarian parenting. 2Reference LTA wave 4 pattern is “Ambiguous Parenting” with all parenting levels elevated.

Supportive parenting was the reference category, and the negative parenting style showed the expected lower levels of self-esteem and self-determination and higher values for emotional symptoms such as dissociation and anxiety/depression for all three regressions (see **Table 11**). This was also true when we compared the pattern of (a) supportive parenting to the pattern of absent parenting and (b) supportive parenting to ambiguous parenting.

## 7 Discussion

The first line of Tolstoy’s novel, “Anna Karenina” declares, “Happy families are all alike; every unhappy family is unhappy in its own way” (74, p. 1), setting the stage for an exploration of different parenting dynamics into the families. Tolstoy’s hidden, and still open to empirical verification presumption is, that the familial dynamics of so-called happy families are very similar. Still, unhappy families are heterogeneous in their structure and processes. In keeping Tolstoy’s assertion in mind, our empirical question is closely connected to the big nature versus nurture topic: Can we understand familial parental dynamics appropriately and then also learn in which direction to possibly change adolescents’ familial circumstances to foster adolescents’ mental health and by doing so change a person in hopes of a happier and more stable mental health life? We have gained various new insights: 1) No person-centred pattern can be assigned to the typical parenting styles. 2) Abused adolescents experience the same parenting style patterns, but they differ in content and stability. Our findings suggest that abuse not only leads to certain patterns but also influences the stability and dynamics of parenting styles.

### 7.1 Person-centered parenting patterns

Following the application of latent person-centred methods for identifying parenting styles (54, 56, 57) longitudinally (LTA) over three waves (LCA), in analyzing our first hypothesis, we add two newer parenting patterns that need more examination in future research: absent and ambiguous parenting to the literature in an increasingly diverse world (75). The “absent” pattern we identified is comparable to the “unsupportive-un-controlling” pattern Teuber et al. (56) or the neglectful pattern Baumrind (18, 19) and Maccoby and Martin (20) described. The “absent” pattern has certain characteristics of parental neglect (43) as it reflects the failure of parents to provide the socio-emotional guidance needed for a child’s emotional health, safety, and well-being. The difference lies in the context of abuse. In the neglectful style of Baumrind (19), the style is characterized by a lack of parental presence and support. In the context of abuse, it can also be characterized by active emotional distancing or hostility. The newly detected style is “ambiguous,” in which all parenting levels were elevated, and is comparable to the inconsistent parenting style, but in contrast to this style also demonstrates high levels of positive parenting. The inconsistent parenting style is characterized by mixed messages and behaviors in similar circumstances whereas the ambiguous style combines high levels of positive and negative parenting.

In further aligning our findings with existing theoretical models of Baumrind’s typology (18, 19), the “absent” parenting style we identified expands neglectful pattern by situating it within a broader sociocultural and non-abusive context, where the absence is often characterized by emotional unavailability rather than hostility. This variation highlights the importance of considering contextual factors such as culture and socioeconomic status that influence parenting practices (e.g., 76, 77). Similarly, the “ambiguous” style offers an extension to the inconsistent parenting style by integrating high levels of both positive and negative parenting, suggesting a more complex interplay of parenting behaviors. This style could potentially provide new insights into existing findings regarding the relationship between attachment and parenting styles (e.g., 78), offering a new perspective on how mixed parental signals influence the development of secure or insecure attachment patterns. Future research could further examine these new styles, to fully integrate their implications into literature.

In the inconsistent parenting style, children can’t anticipate their parents’ reactions to situations. In the APQ, statements such as “the punishment your parents give depends on their mood” are common. The emphasis is on being able to talk one’s parents out of a threatened punishment or on parents not being strict in enforcing their punishment. This style leads to uncertainty and anxiety. The ambiguous pattern, on the other hand, does not focus on anticipating a reaction or specifically on punishment. This new pattern demonstrates that positive and negative parenting styles can overlap. Children experience warmth, closeness and support, but at the same time are also subjected to high levels of control and discipline. This pattern is contradictory rather than inconsistent and might lead to both attachment and mistrust. As far as we know, a person-centered study that looked at the overlap of these styles has so far not been conducted. We therefore proposed that investigating this pattern might provide information about the conditional parenting style, where parents use attachment to condition children’s behaviors. This parenting style is associated with introjected self-regulation, depressive symptoms, and contingent self-esteem, as meta-analysis demonstrates (79). As Rossman and Rea (80) have shown, children in families where parents fluctuate between positive and negative behavior patterns due to stress, abuse, or their insecurities can receive conflicting signals, which can lead to behavioral problems and emotional difficulties. The study emphasizes the importance of consistent and supportive parenting practices for the healthy development of children, especially in stressful family situations. These results are important, as meta-analysis (see for example, 81) finds conflicting results when looking at rigid categorized parenting styles.

### 7.2 Differences between abused and nonabused participants

Generally speaking, abused adolescents experience significantly worse negative parenting styles than nonabused adolescents and their positive parenting styles are also less positive. Supportive parenting remains less stable and is less pronounced in abused adolescents than in nonabused adolescents. The ambiguous pattern differs between the abused/nonabused groups in that the abused adolescents clearly experience an inconsistent parenting style, but the positive dimensions are also more pronounced. Abused adolescents are more likely to be exposed to even more inconsistent behavior and lack of supervision. For nonabused adolescents on the other hand, the ambiguity is stronger in the direction of involvement, indicating a controlling parenting style, similar to the tiger style of Kim et al. (55). The pattern of absent parenting is more pronounced in abused adolescents and includes more negative styles such as Poor Supervision and Authoritarian. We think it is important to consider that no distinct separation of the original parenting styles was found, demonstrating that latent parenting patterns are more suitable for mapping the complex reality of parenting. According to these insights, we must use a more complex approach, using a blend of different parenting styles that also differ in terms of the status of abuse.

In our study with a representative Swiss sample of 1,709 adolescents, about half of the participants were found in these two new patterns at all three waves, reliably detected from early to middle adolescence, with an increase in ambiguous style from Waves 1 to 3. Therefore, the absent and ambiguous parenting styles appear to play a key role in adolescents with and without experience of abuse.

Related to the development of the identified four parenting styles over time (H2) and in line with previous longitudinal latent person-centred research on parenting styles (56, 57), the identified patterns developed diversely. Where the supportive pattern for abused adolescents decreased by a large margin, we noticed high stability among nonabused adolescents. For the absent pattern among abused adolescents, we found considerably higher stability than for the negative pattern of the abused adolescents. On the contrary, similar to Teuber et al. (56), we identified a remarkable decline for the nonabused adolescents in the absent pattern from Wave 1 to Wave 3. Even if the number of the patterns identified were the same (configural invariance) for both, the nonabused and the abused adolescents, metric invariance was not shown, suggesting that abused adolescents reported more negative parenting and less positive parenting than nonabused adolescents.

We additionally highlight that both newly identified parenting styles, the absent and ambiguous styles, are present among abused and nonabused participants, with the difference of higher negative parenting levels in abused participants. Of abused participants who reported in Wave 1 that their parents were supportive, a quarter reported that their parents were no longer supportive at Wave 3, compared to just 5% of the nonabused sample. The number of abused youths who reported ambiguous parenting at Wave 3 increased from Wave 1 to Wave 2, and 91.8% of them reported negative, absent, or ambiguous styles in Wave 3. The high stability of the absent parenting style in abused adolescents suggests that in future research, in addition to the positive or negative patterns observed in this population so far, it is essential to include more information about this parenting style and the associated mental health outcomes over time in adolescents who have experienced abuse, as Gu et al. (42) did. The supportive parenting style was characterized by high involvement and positive parenting and very low probabilities of negative parenting indicators in wave 1 for both abused and nonabused adolescents. Through the waves, negative parenting indicators gained some momentum (although still low) for the abused adolescents in the supportive style, only to decline again in the final wave. Still, mental health outcomes in adolescents were very positive for this type of parenting style, demonstrating the protective factor of a supportive parenting style. This finding emphasizes that a positive relationship can recover from negative life events such as Covid-19. Concerning attachment theory, our results demonstrate that early attachment experiences are formative, but also changeable. The characteristics of the parenting styles that we examined changed slightly within the individual groups over time, but were still distinct (82).

Concerning mental health, adolescents in both subgroups assigned to the supportive parenting pattern had the lowest levels of internalizing problems (H3) and highest levels of self-determination (83) and self-esteem (13, 84). Conversely, the negative, absent, and ambiguous profiles showed the opposite trends, with adolescents in the negative pattern having significantly lower self-esteem and self-determination levels than those in the positive pattern. Similarly, they demonstrated higher levels of emotional distress, including dissociation and anxiety/depression. The absent parenting pattern also involved less self-esteem and self-determination than the ambiguous pattern, but there was no difference in dissociation and anxiety/depression. These insights from our study have important educational implications for teacher support (85) and parental involvement (86). Policymakers could also consider them in developing policies and approaches that will help to protect abused adolescents’ mental health. Delineating the four identified unique latent patterns may lead to the design of more modular or combined treatments or counseling supports that address concomitant parenting-style-specific problems.

### 7.3 Contextual interpretation

Based on the findings of Vazsonyi et al. (87), some of the parenting practices are thought to have a universal effect on adolescent development, particularly regarding the development of internalizing and externalizing behaviors. However, the specific way in which these practices are perceived, and work depends on the cultural context. For example, the Swiss context is characterized by multilingualism, is mostly determined by individualistic values such as self-discipline, performance, punctuality and is shaped by long-term migration processes. Our findings are therefore particularly useful for cultural contexts such as Northern Europe, the USA and Canada, in which individualistic values such as autonomy and self-realization, achievement orientation and school success are paramount, and which are furthermore characterized by multiculturalism and cultural diversity. Distinguishing between these universal mechanisms and cultural specificities is beyond the scope of our study, but future studies conducted across cultures are important in this regard, as they provide a useful framework for considering the broader generalizability of our findings. Furthermore, the COVID-19 pandemic has had an impact on family life and parenting practices. Studies show that the pandemic has led to increased stress and psychological strain among parents, which can have a negative impact on parenting behavior. For example, the Swiss Federal Office of Public Health (88) reports that children, adolescents and young adults were significantly more affected by the psychological consequences of the pandemic than other age groups, especially those who were already under stress before the crisis (89). These reports indicate that the pandemic may have led to an increase in ambivalence and absence in parenting patterns, while the lifting of restrictions in some families might have contributed to a return to supportive behavior.

### 7.4 Limitations

Swiss data on parenting styles align with other international data (90); however, further work is needed to understand the composition of the ambiguous and absent patterns styles experienced by youth. Another limitation could be stated regarding the largely unbalanced sample sizes for the abused and the nonabused groups, which may have potentially led to less reliable comparisons between groups. Hopefully, the abused may represent a small percentage of the general population, making it challenging to ensure balanced samples in statistics (e.g., 91, 92). Also, Wave 1 was conducted at the start of the COVID-19 pandemic, and Waves 2 and 3 occurred after its conclusion. Studies have indicated that the initial phases of the pandemic had far more negative consequences for mental health than the later phases (93). Additionally, we had just one data source, adolescents’ perceptions, which can limit the results. This approach does not consider the perspectives of other stakeholders such as parents, teachers or peers who could provide a broader context or alternative viewpoints. Nevertheless, it can be stated that the perspective of young people is very important and therefore a triangulation of different perspectives is important for future research. It is possible that being abused affects the parenting style for supportive parents, but we cannot determine causal direction from this data. For example, the number of adolescents who report abuse and have supportive parents declines from wave 1 to wave 3, while the frequencies are stable for other parenting styles. Several plausible theories may be worth exploring in further studies.

Furthermore, we did not take into account the possible parenting differences of mothers or fathers (94) or parent-child discrepancies in parental monitoring reports (95). Independent data collection from both parents (58) and youth using the APQ shows consistency in parenting style and developmental psychology studies (Esposito, 2016), suggesting that youth perception is the most important variable whereas others compare youth to parent assessments (58). Finally, if we conceptualize parenting styles as dynamic and person-centered, these ways of interacting can be understood as protective or detrimental factors in the lives of children and adolescents and therefore as multifaceted. Our study introduces new parenting patterns, namely absent and ambiguous, which emphasizes the need for a more complex approach to understanding parenting styles. Future research should focus on exploring the composition and characteristics of these patterns in more detail. We also note that it is important to consider parenting styles in terms of the concept of resilience, which we were not able to do in this paper and believe that our findings show that future research should focus on understanding parenting styles as resilience factors.

### 7.5 Conclusions

All in all, our study offers significant insights into the dynamic complexities of parenting styles, particularly in the context of abuse, by introducing absent and ambiguous parenting patterns. These patterns underscore the importance of understanding familial dynamics and their impact on adolescents’ mental health. Our findings highlight that while supportive parenting serves as a protective factor, the negative, absent, and ambiguous patterns contribute to significantly lower levels of self-esteem and self-determination, especially in the case of abuse. Parents need to be sensitized to the particular importance of supportive parenting as well as the risk factor they impose on their children if they demonstrate negative, absent or ambiguous parenting patterns. Practical findings, furthermore, on parenting styles need to be considered more broadly, as focusing on individual styles might be too narrow. To date, no law in Switzerland prohibits physical violence against children; this must be implemented as a signpost. Policymakers can develop low-threshold services aimed at raising awareness, providing support and furthermore, the school can act as a safe space that uncovers such situations and promotes the socio-emotional development of its children and young people. To close with Tolstoy’s novel, we found more differences in negative forms of parenting than positive forms.

## Data Availability

The raw data supporting the conclusions of this article will be made available by the authors, without undue reservation.
